# Prophylactic Enoxaparin Dosing After Bariatric Surgery: A Systematic Review and Meta-Analysis of Pharmacodynamic Efficacy and Safety

**DOI:** 10.1007/s11695-026-08825-9

**Published:** 2026-07-21

**Authors:** Czara Kennedy, Mohammed Al Azzawi, Matthew Davey, Julie Therese Clifford, Waqas Butt, Noel Donlon, Karen Murphy, Naomi Fearon, Helen Heneghan

**Affiliations:** 1 Department of Upper GI and Bariatric Surgery, St. Vincent’s University Hospital, Dublin, Ireland; 2 Irish Surgical Research Collaborative, Royal College of Surgeons in Ireland, Dublin, Ireland; 3 Irish Surgical Research Collaborative, Royal College of Surgeons in Ireland, Dublin, Ireland; 4 Department of Haematology, St. Vincent’s University Hospital, Dublin, Ireland

## Abstract

**Introduction:**

Venous thromboembolism (VTE) remains a leading cause of preventable morbidity and mortality following metabolic bariatric surgery (MBS). Low-molecular-weight heparin (LMWH) is widely used for thromboprophylaxis; however, the optimal prophylactic enoxaparin dose in patients with obesity remains uncertain. Anti-factor Xa (anti-Xa) activity is commonly used as a surrogate marker of pharmacological efficacy.

**Methods:**

A systematic review and meta-analysis was conducted in accordance with PRISMA guidelines and registered with PROSPERO. PubMed, EMBASE, the Cochrane Library, and ClinicalTrials.gov were searched from inception to 31 March 2026. Studies evaluating prophylactic enoxaparin dosing in patients with obesity undergoing MBS, with reported anti-Xa levels, were included. Random-effects meta-analyses compared lower (30–40 mg) versus higher (60 mg) enoxaparin regimens with respect to attainment of prophylactic anti-Xa levels with secondary outcomes including mean anti-Xa activity, VTE and bleeding event rates.

**Results:**

Ten studies comprising 1,069 enrolled patients, of whom 1,016 contributed to the primary meta-analysis, met the inclusion criteria. There was no significant difference in the proportion of patients achieving prophylactic anti-Xa levels between higher and lower dose regimens (risk ratio 1.05, 95% CI 0.91–1.20; I² = 58.2%, *p* = 0.0104). Higher doses were associated with significantly increased mean anti-Xa levels (mean difference 0.13 IU/mL, 95% CI 0.05–0.21; *p* = 0.001).

**Conclusion:**

Higher prophylactic enoxaparin doses increase anti-Xa levels but do not improve attainment of prophylactic targets compared with standard dosing following MBS. In a post-hoc, exploratory, hypothesis-generating subgroup analysis, BMI-adjusted dosing strategies demonstrated comparable pharmacodynamic efficacy to fixed higher doses; this finding is not pre-specified and should not be used to support definitive conclusions. High-quality multicentre randomised trials are required to define optimal thromboprophylaxis strategies in this high-risk population.

**Supplementary Information:**

The online version contains supplementary material available at https://doi.org/10.1007/s11695-026-08825-9.

## Key Points

Higher enoxaparin doses increase anti-Xa levels after metabolic bariatric surgery.

Target anti-Xa attainment is similar with standard and higher dosing regimens.

Bleeding and VTE event rates were too low across included studies to permit formal meta-analysis or comparative safety conclusions.

Robust randomised trials are needed to guide thromboprophylaxis practice.

**Supplementary Information:**

The online version contains supplementary material available at https://doi.org/10.1007/s11695-026-08825-9.

## Introduction

Obesity is both an independent and additive risk factor for deep vein thrombosis (DVT) and pulmonary embolism (PE), and all patients undergoing metabolic bariatric surgery (MBS) fall under moderate to high risk for VTE development. Obesity confers a hypercoagulable state due to elevated coagulation factors (VII, VIII, fibrinogen, von Willebrand factor, and PAI-1), compounded by poor glycaemic control, dyslipidaemia, inflammation, endothelial dysfunction, and the surgical procedure itself [1–4]. 

Venous thromboembolism (VTE) continues to be the leading cause of mortality following MBS. Studies have shown that over 80% of VTEs occur post-discharge and within 30 days of surgery, providing the rationale for extended prophylaxis [5–8]. Despite advances in MBS, no consensus exists on the optimal thromboprophylaxis strategy [9, 10]. The ASMBS recommends extended pharmacoprophylaxis only for select high-risk patients but does not specify dose, duration, or agent [8, 11, 12]. Likewise, the 2018 NICE guidelines contain limited guidance on pharmacological agent or dosing for patients undergoing MBS [13]. 

Low molecular weight heparin (LMWH) is established for VTE prevention and exerts its effect through Factor Xa inhibition via antithrombin, allowing monitoring with anti-Xa serum levels. Peak levels are reached 3–5 h post-administration [14–17]. Anti-Xa monitoring is recommended when total body weight exceeds 150 kg. The prophylactic anti-Xa range of 0.2–0.4 IU/mL is widely cited in clinical practice guidelines as the target for LMWH prophylaxis in high-risk surgical patients undergoing MBS, based on expert consensus and pharmacokinetic data from general surgical and medical populations. It should be noted that no prospective study has directly demonstrated that achievement of this anti-Xa range reduces symptomatic VTE specifically in patients undergoing MBS. Anti-Xa activity is therefore used throughout this review as a pharmacodynamic surrogate endpoint, and its validity as a predictor of clinical VTE prevention in this specific population remains an important unresolved question [16, 18, 19]. 

The efficacy and safety of LMWH in patients with obesity remains unclear, as they are frequently excluded from randomised trials. Existing studies are limited by small sample sizes, heterogeneous dosing regimens, and a lack of control groups, leaving clinicians uncertain whether standard or weight-adjusted doses are required [20]. This review examines published data on prophylactic enoxaparin dosing in patients undergoing MBS, using anti-Xa levels as the primary pharmacodynamic endpoint.

## Methods and Materials

This review was performed according to the Preferred Reporting Items for Systematic reviews and Meta-Analyses (PRISMA) Statement [21]. It was registered with PROSPERO as CRD420251104111. Ethical approval was not required from the local institutional review board, as this study utilised data exclusively from previously published sources.

A comprehensive search was conducted using PubMed, EMBASE, the Cochrane Library, and ClinicalTrials.gov were searched from inception to 31 March 2026. Grey literature was searched via OpenGrey and the WHO ICTRP. Conference proceedings from the ASMBS annual meeting were reviewed, and authors of completed but unpublished trials were contacted for data; no additional datasets were obtained. Reference lists of included studies and relevant systematic reviews were hand-searched. No restrictions on language, publication type, or year were applied.

The PubMed search strategy is presented below; full database-specific strings are provided in Supplementary Appendix 1:

(“Bariatric Surgery“[Mesh] OR “bariatric surgery” OR “obesity surgery” OR “Roux-en-Y” OR “gastric bypass” OR “sleeve gastrectomy”) AND (“Low-Molecular-Weight Heparin“[Mesh] OR “LMWH” OR “enoxaparin”) AND (“Factor Xa“[Mesh] OR “anti-Xa” OR “anti-factor Xa” OR “anti-Xa level*”) AND (“Venous Thromboembolism“[Mesh] OR “VTE” OR “deep vein thrombosis” OR “DVT” OR “pulmonary embolism” OR “thromboprophylaxis”)

### Study Selection

All titles were initially evaluated, duplicates were removed, and suitable abstracts were extracted for full-text screening based on the inclusion and exclusion criteria. Two independent reviewers (C.K. and M.A.) screened the titles and abstracts of studies. Conflicts were resolved by a third reviewer (M.D.). Full-text articles of potentially eligible studies were retrieved for detailed evaluation based on predefined inclusion and exclusion criteria.

## Inclusion and Exclusion Criteria

### Inclusion Criteria

- Patients with obesity undergoing metabolic bariatric surgery.
- Studies evaluating two different prophylactic doses of enoxaparin in the same patient population, enabling direct dose comparison, with anti-factor Xa activity reported as a pharmacodynamic endpoint. Single-arm studies were excluded as they do not permit between-dose comparison.
- Prophylaxis efficacy measured by anti-factor Xa levels.

## Exclusion Criteria

- Patients did not undergo metabolic bariatric surgery.
- Post-operative pharmacological regimen did not include two arms of enoxaparin.

## Data Extraction and Statistical Analysis

The following data were retrieved from the selected publications using a standardised extraction form to find potentially relevant articles: journal, author, year, published country, number of patients per arm, specific treatment applied, mean anti-Xa level, proportion of patients that achieved prophylaxis as measured by anti-Xa levels and VTE/bleeding event rates. Duplicates were erased and the discrepancies clarified.

### Outcomes

For the purposes of this review, the primary outcome was efficacy, defined as the proportion of patients achieving adequate pharmacological prophylaxis as determined by anti–factor Xa activity levels. Secondary outcomes included the comparison of mean anti–factor Xa levels and mean BMI between treatment groups, as well as the incidence of VTE and bleeding events. Bleeding complications were categorised as major or minor according to the criteria of the International Society on Thrombosis and Haemostasis (ISTH).

### Statistical Analysis

Descriptive statistics characterised patient demographics, treatment duration, and dosing regimens. Dichotomous outcomes (proportion achieving prophylaxis, VTE and bleeding event rates) and continuous outcomes (anti-Xa levels and BMI) were analysed accordingly. Pooled mean BMI values were calculated using sample size-weighted averages (IBM SPSS Statistics, Version 29.0).

A random-effects meta-analysis was conducted using the meta package (version 4.3.2, R Foundation for Statistical Computing) to compare the proportion of patients achieving prophylactic anti-Xa levels between higher and lower enoxaparin regimens. Risk ratios (RRs) with 95% confidence intervals (CIs) were calculated using the Mantel-Haenszel method with a DerSimonian-Laird random-effects model and Hartung-Knapp adjustment. Heterogeneity was assessed using I², τ², and Cochran’s Q. Publication bias was evaluated visually with a funnel plot and statistically using Egger’s and Begg’s tests. A post-hoc exploratory subgroup analysis compared BMI-adjusted versus fixed-dose regimens; this was not pre-specified in the PROSPERO protocol and should be considered hypothesis-generating only. A post-hoc sensitivity analysis excluding Vaughns 2020 generated an adult-only pooled estimate.

Trial Sequential Analysis (TSA) was performed using TSA software (version 0.9.5.10 Beta, Copenhagen Trial Unit), applying two-sided alpha of 5%, power of 80%, and O’Brien-Fleming spending functions, with heterogeneity correction (D²=0.71) [22]. 

## Assessment of Risk of Bias

Methodological quality of the two randomised controlled trials was assessed using the Cochrane Risk of Bias 2 (RoB 2) tool, which evaluates five domains: bias arising from the randomisation process, deviations from intended interventions, missing outcome data, measurement of outcomes, and selection of reported results [23]. Each domain was rated as low risk, some concerns, or high risk, with an overall risk of bias judgement assigned accordingly [24]. Observational study quality was assessed using the Newcastle-Ottawa Quality Assessment Scale (NOS) [25, 26]. The certainty of evidence for the primary outcome was assessed using the GRADE framework, with ratings downgraded for risk of bias, inconsistency, and imprecision as appropriate [27]. 

## Results

### Literature Search and Study Characteristics

**Fig. 1:**
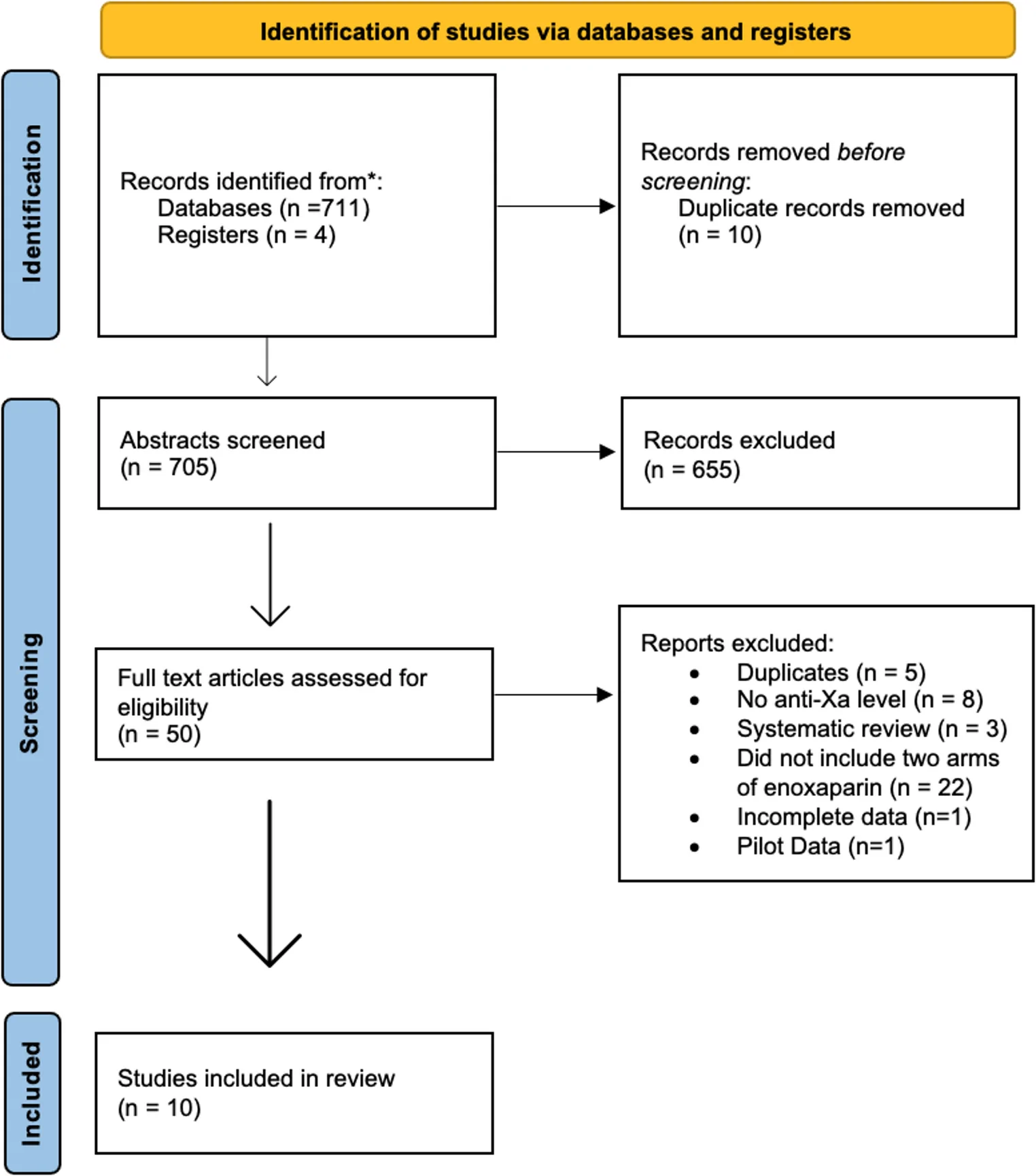
PRISMA Flowchart

A total of 715 records were identified through database (*n* = 711) and registry searching (*n* = 4). Following removal of 10 duplicate records, 705 abstracts were screened, of which 655 were excluded. Fifty full-text articles were assessed for eligibility; 40 were excluded for the following reasons: duplicates (*n* = 5), no anti-Xa level reported (*n* = 8), systematic reviews (*n* = 3), did not include two arms of enoxaparin (*n* = 22), incomplete data (*n* = 1), and pilot data (*n* = 1). Ten studies met the predefined inclusion criteria and are included in this review. Figure 1 depicts the PRISMA flowchart. Of the ten included studies, two were randomised controlled trials, two were retrospective cohort studies, and six were prospective cohort studies. All studies reported the proportion of patients achieving prophylaxis as measured by anti-Xa levels whilst receiving prophylactic enoxaparin. Publication dates ranged from 2007 to 2024.

### Patient and Study Characteristics

In total, 1,069 patients were enrolled across the ten studies included in this systematic review. The characteristics of all ten studies are presented in Tables 1 and 2. Across the studies included, there were 280 male patients (26.2%) and 789 female patients (73.8%). Of these, 1,016 patients contributed to the primary meta-analytic forest plot; the remaining 53 patients were enrolled but did not have anti-Xa levels recorded within the required sampling window and were therefore not included in the pooled analysis. Steib et al. (2015) employed a three-arm design; only the 4,000 IU OD and 6,000 IU OD arms were included in this analysis as these represent the direct dose comparison of interest. The 2 × 4,000 IU BD arm (*n* = 47 per protocol) was excluded as it reflects a dosing frequency comparison rather than dose escalation. The timing of anti-Xa level sampling was consistently reported across included studies; samples were collected between 3 and 5 h following the second or third dose. Among the included studies, the most frequently evaluated enoxaparin regimens were 40 mg BD and 60 mg BD. Across all cohorts, 433 patients received a 40 mg BD regimen and 374 patients received a 60 mg BD regimen. Once-daily dosing was evaluated in 103 patients receiving 40 mg OD and 95 patients receiving 60 mg OD. A smaller subset of 11 patients received a lower 30 mg BD regimen.

### Duration of Treatment

For the studies with reported durations, the treatment length ranged from 3 days in the Vaughns et al. study to a longer range of 14–28 days in the Karas et al. study. Two other studies, Borkgren-Okonek et al. and Steib et al., both reported a mean treatment duration of 10 days.

### Descriptive Statistics for Patient Body Mass Index

The pooled mean BMI for the Enoxaparin 30/40 mg group was 44.82 kg/m² (SD = 2.60 kg/m²), while the pooled mean BMI for the Enoxaparin 60 mg group was 49.26 kg/m² (SD = 7.09 kg/m²), based on parametric data from nine paired studies. A paired samples t-test revealed that the difference between the two groups was not statistically significant (t = −1.75, df = 8, *p* = 0.12, two-sided).

### Trial Sequential Analysis

Trial Sequential Analysis for the primary outcome of anti-Xa target attainment demonstrated a required information size of 1,570 patients to detect a relative risk reduction of 17.5% with 80% power and two-sided alpha of 5%, incorporating a heterogeneity correction of 70.78% (D² = 0.71). The accrued sample of 1016 patients, represents approximately 65% of the required information size. The cumulative Z-curve did not cross either the superiority or futility monitoring boundaries at any point during the sequential analysis, remaining within the inconclusive zone throughout. (Appendix - Supplementary Fig. 1)

### Primary Endpoint

**Fig. 2:**
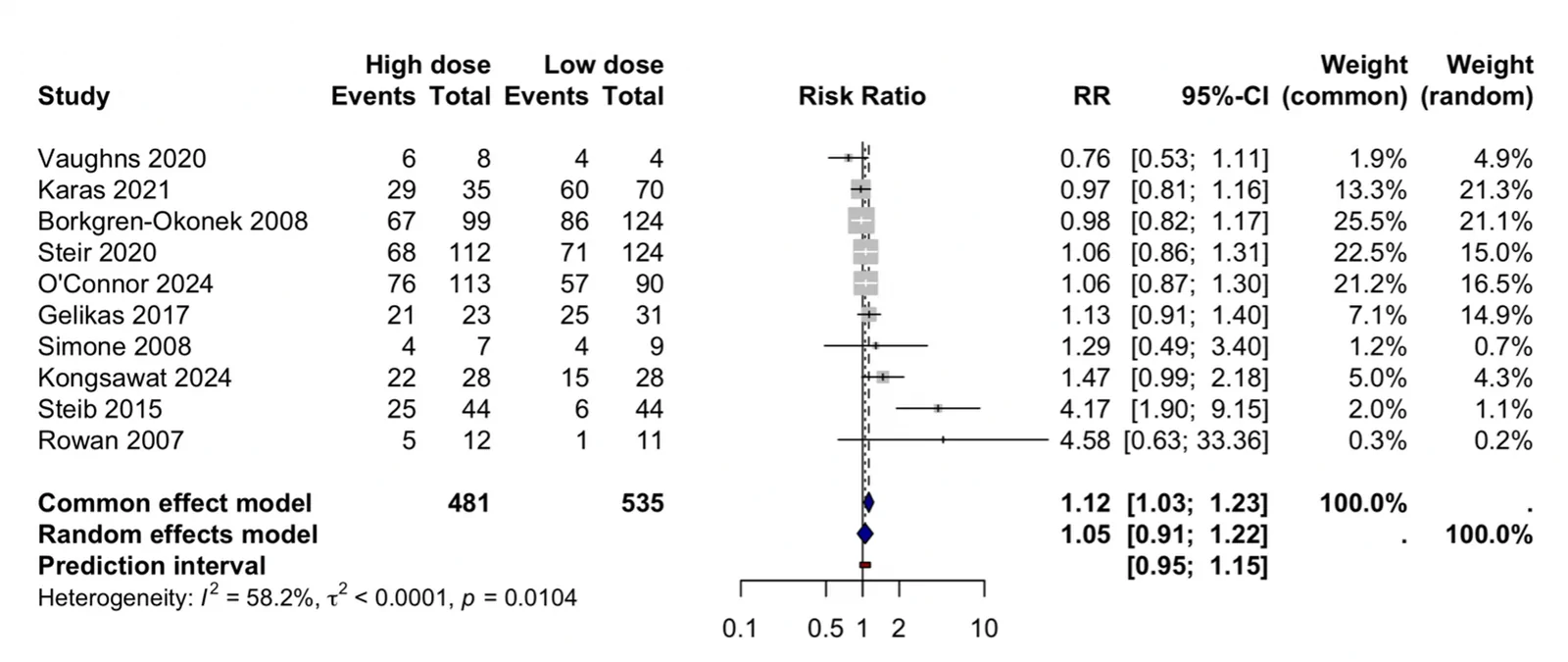
Proportion Achieving Prophylaxis as measured by Anti-Xa levels

The proportion of patients that achieved prophylaxis, mean anti-Xa levels, rates of VTE and bleeding can be seen in Table 3.A meta-analysis of ten studies, including 535 patients in the lower-dose enoxaparin group (30 or 40 mg) and 481 patients in the higher-dose group (60 mg), evaluated the efficacy of different prophylactic enoxaparin regimens in achieving target anti-Xa concentrations (Fig. 2). The pooled risk ratio was 1.05 (95% CI 0.91–1.22; I² = 58.2%, *p* = 0.0104), with a prediction interval of 0.95–1.15. No statistically significant difference was observed between higher and lower dosing strategies in the proportion of patients achieving prophylactic anti-Xa targets. However, the confidence interval of 0.91–1.22 is wide and fully consistent with clinically meaningful benefit or harm in either direction; this result does not support a conclusion of equivalence between dosing regimens. The Hartung-Knapp adjustment was applied to account for uncertainty in the between-study variance estimate; with ten studies contributing to the primary analysis, the pooled estimate should be interpreted with particular caution. Substantial heterogeneity was observed across included studies.

**Fig. 3:**
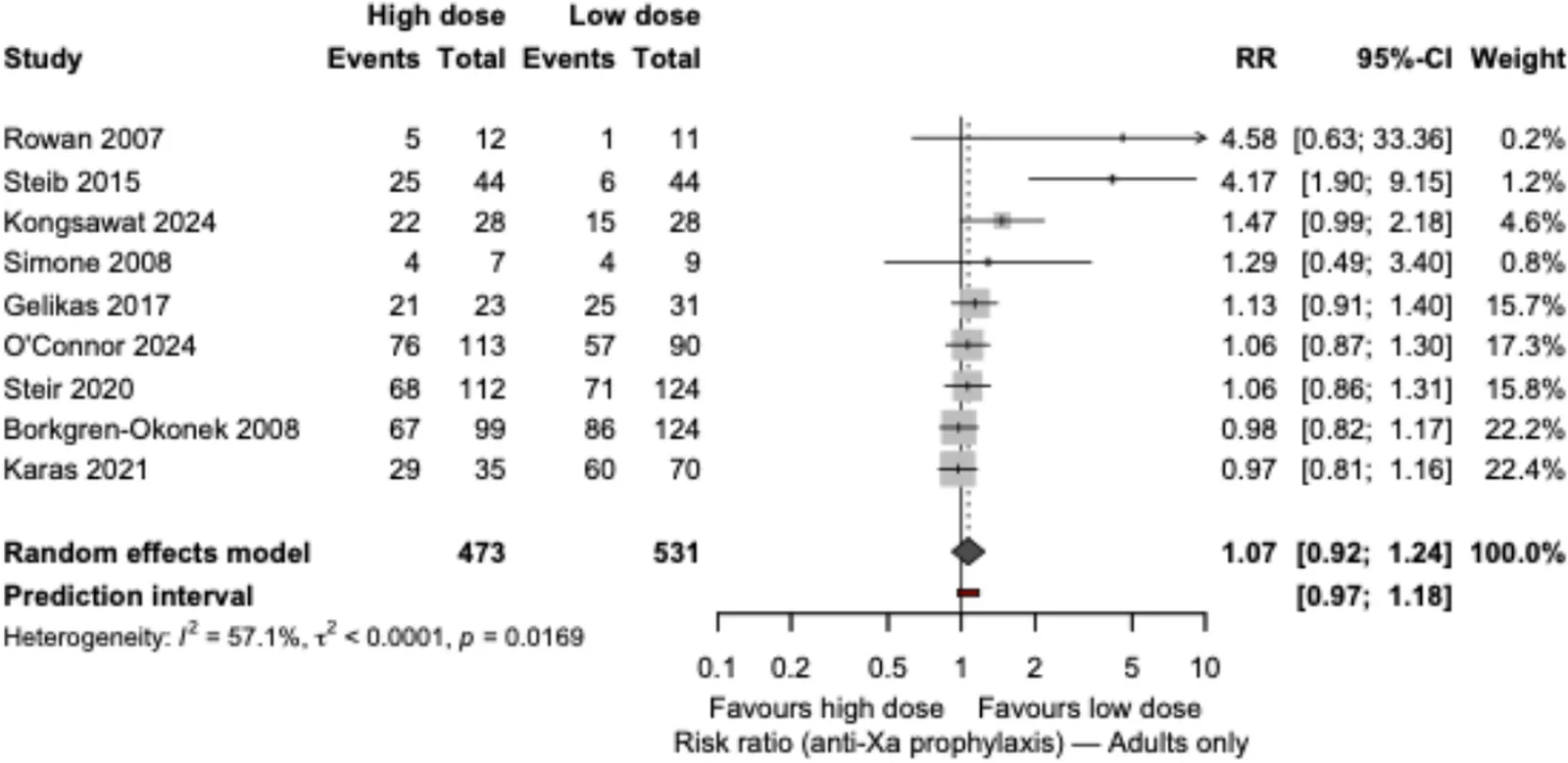
Proportion of patients achieving prophylactic anti-Xa levels with higher versus lower dose enoxaparin regimens in adult patients undergoing metabolic bariatric surgery (Vaughns 2020 excluded)

In a post-hoc sensitivity analysis excluding Vaughns 2020, nine studies comprising 531 lower-dose and 473 higher-dose patients were analysed (Fig. 3). The pooled risk ratio was 1.07 (95% CI 0.92–1.24; I² = 57.1%, *p* = 0.0169), with a prediction interval of 0.97–1.18, consistent with the primary analysis in both direction and magnitude.

**Fig. 4:**
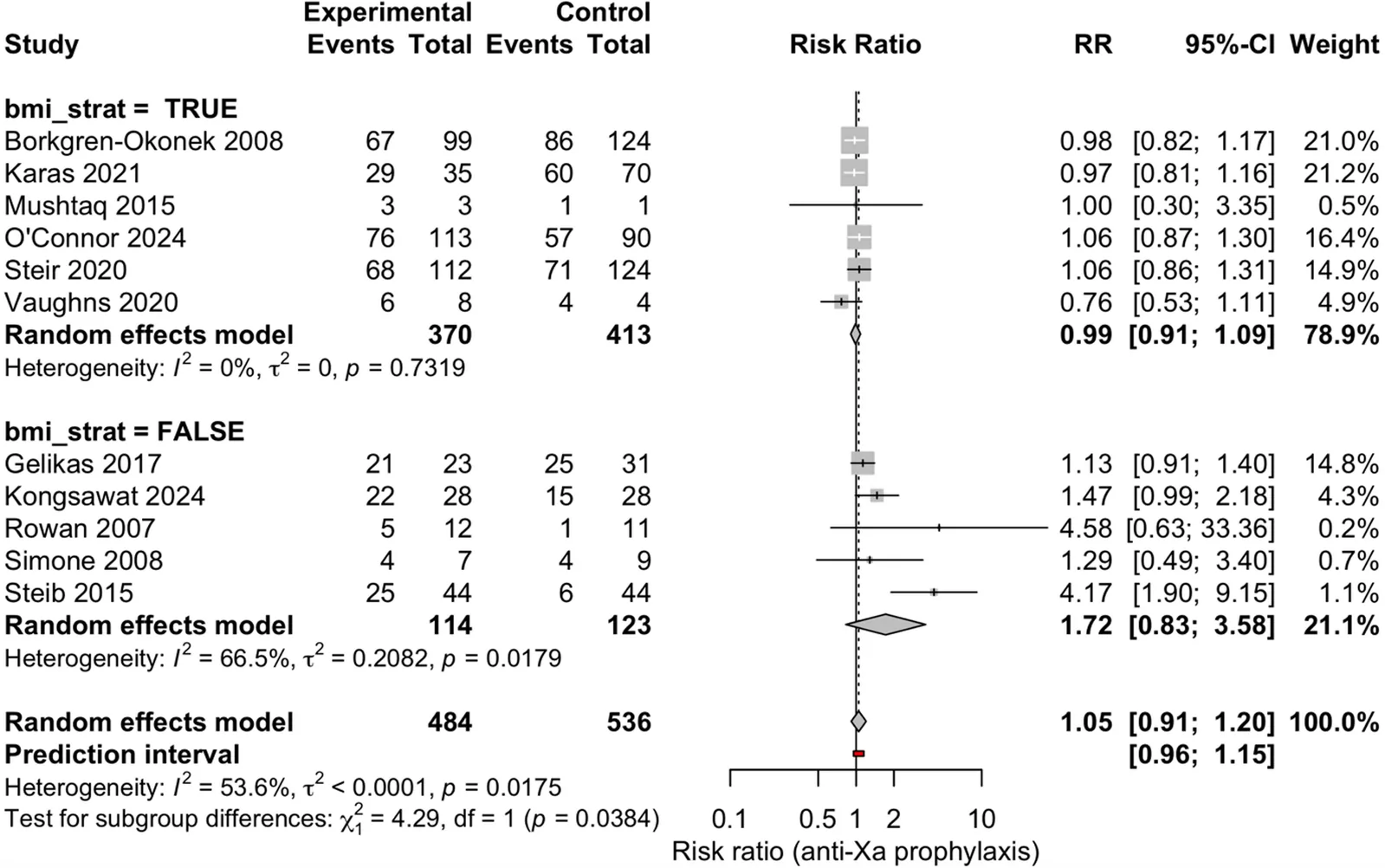
Effect of BMI-adjusted versus fixed-dose enoxaparin regimens on attainment of prophylactic anti-Xa levels

A subgroup analysis was performed to evaluate whether BMI-stratified dosing influenced the proportion of patients achieving prophylactic anti-Xa levels (Fig. 4). Among studies that adjusted enoxaparin dose according to BMI, the pooled risk ratio was 0.99 (95% CI 0.91–1.09), with no evidence of heterogeneity (I² = 0%, *p* = 0.73), indicating comparable efficacy between higher and lower doses. In contrast, non–BMI-stratified studies demonstrated a pooled risk ratio of 1.72 (95% CI 0.83–3.58) with substantial heterogeneity (I² = 66.5%, *p* = 0.018), suggesting variable findings and a potential trend favouring higher doses. The test for subgroup differences was statistically significant (χ² = 4.29, df = 1, *p* = 0.038), indicating that the relationship between enoxaparin dose and attainment of prophylactic anti-Xa levels differed according to dosing strategy. This analysis was post-hoc and exploratory, was not pre-specified in the PROSPERO protocol, and should be considered hypothesis-generating only. It should not be used to support definitive conclusions regarding the superiority or equivalence of BMI-adjusted dosing strategies.

### Mean Difference in Anti-Xa Levels Between Enoxaparin 40 mg and 60 mg Dose Groups

**Fig. 5:**
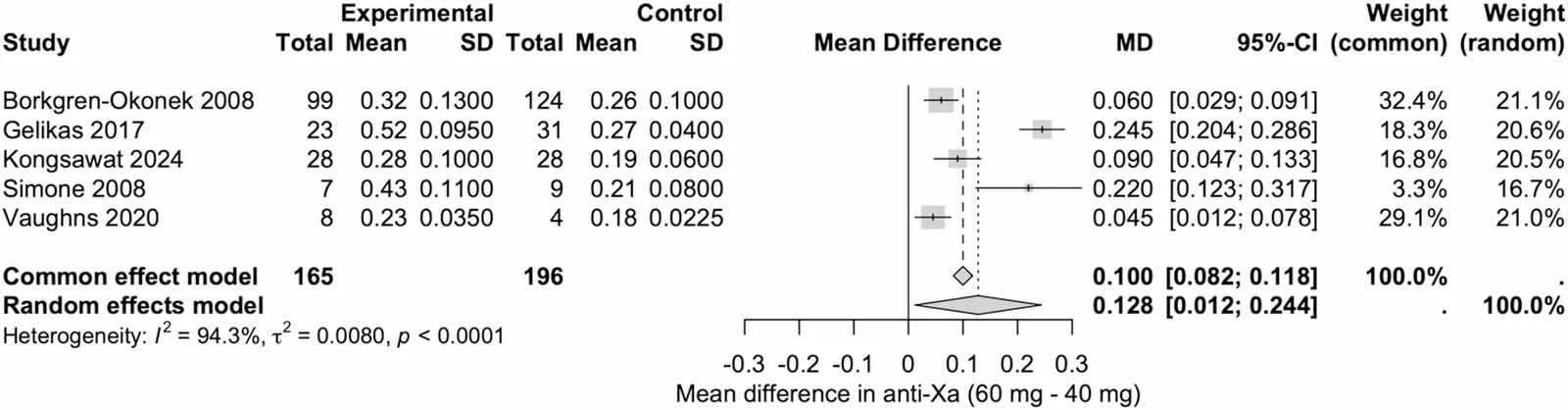
Mean Difference in Anti-Xa Levels Between Enoxaparin 40 mg and 60 mg Dose Groups

A meta-analysis of five studies was performed to compare mean anti-Xa levels between patients receiving Enoxaparin 40 mg and those receiving 60 mg daily. The combined analysis included 196 participants in the 40 mg group and 165 in the 60 mg group. Estimated means and standard deviations from Gelikas et al. and Vaughns et al. were incorporated to ensure their inclusion. The pooled mean difference in anti-Xa levels (40 mg *versus* 60 mg) was 0.13 IU/mL (95% CI 0.05 to 0.21; *p* = 0.001), indicating significantly higher anti-Xa levels with the 60 mg regimen (Fig. 5). Four studies (Gelikas et al., Kongsawat et al., Simone et al., and Vaughns et al.) demonstrated statistically significant increases in anti-Xa activity with the higher dose. In contrast, Borkgren-Okonek et al., the largest study, showed a smaller, non-significant difference between the two dosing groups.

**Fig. 6:**
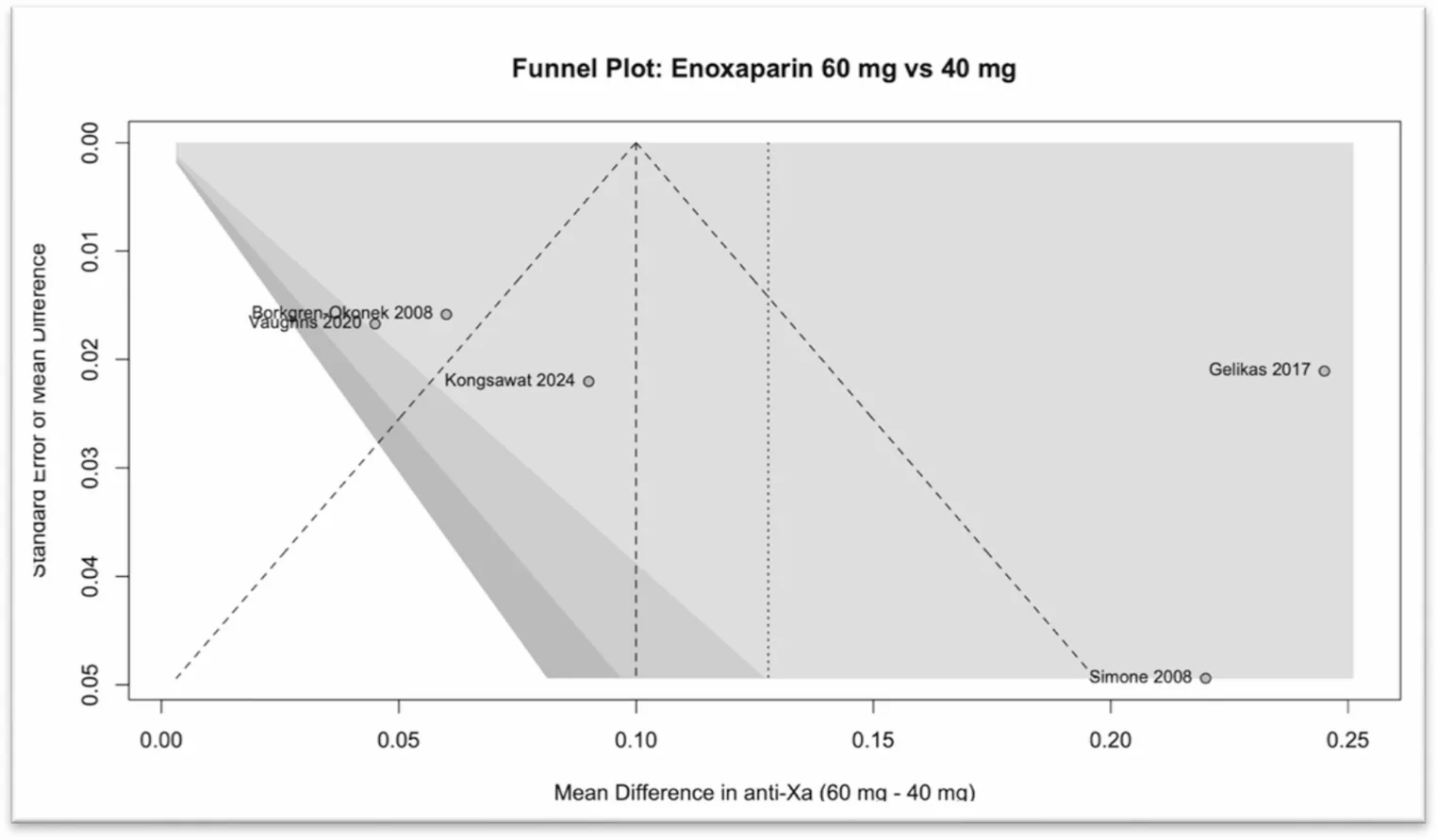
Funnel plot assessing publication bias in studies comparing mean anti-Xa levels between Enoxaparin 60 mg and 40 mg groups

Substantial heterogeneity was observed among studies (I² = 94.3%, τ² = 0.0080, *p* < 0.0001). The funnel plot showed mild asymmetry, with smaller studies tending to report larger mean differences in anti-Xa levels (Fig. 6). Egger’s test indicated no significant publication bias (*p* > 0.05), suggesting that the observed asymmetry likely reflects between-study variability rather than selective reporting. The I² of 94.3% represents extreme statistical heterogeneity and substantially limits the interpretability of the pooled mean difference estimate. At this level of heterogeneity, the pooled result should be considered an unreliable summary of the constituent studies rather than a stable estimate of the true treatment effect. Potential sources include differences in dosing frequency (OD versus BD), anti-Xa assay methodology, patient BMI distribution, and sampling time points; however, the small number of contributing studies (*n* = 5) precluded formal meta-regression to explore these drivers. This analysis should therefore be interpreted with caution and regarded as exploratory.

### Once-Daily (OD) Dosing Subgroup (40 mg OD vs. 60 mg OD)

The once-daily dosing subgroup included the studies by *Gelikas et al.* and *Kongsawat et al.* The pooled mean difference in anti-Xa levels was − 0.17 IU/mL (95% CI − 0.32 to − 0.02; *p* = 0.03), indicating significantly higher anti-Xa levels with the 60 mg dose. Substantial heterogeneity was observed (τ² = 0.01; χ² = 25.84, df = 1, *p* < 0.00001; I² = 96%).

### Twice-Daily (BD) Dosing Subgroup (40 mg BD vs. 60 mg BD)

The twice-daily dosing subgroup included the studies by *Borkgren-Okonek et al.*, *Simone et al.*, and *Vaughns et al.* The pooled mean difference was − 0.13 IU/mL (95% CI − 0.29 to 0.04; *p* = 0.13), which was not statistically significant. Heterogeneity remained high (τ² = 0.01; χ² = 11.26, df = 2, *p* = 0.004; I² = 82%).

### Subgroup Comparison

**Fig. 7:**
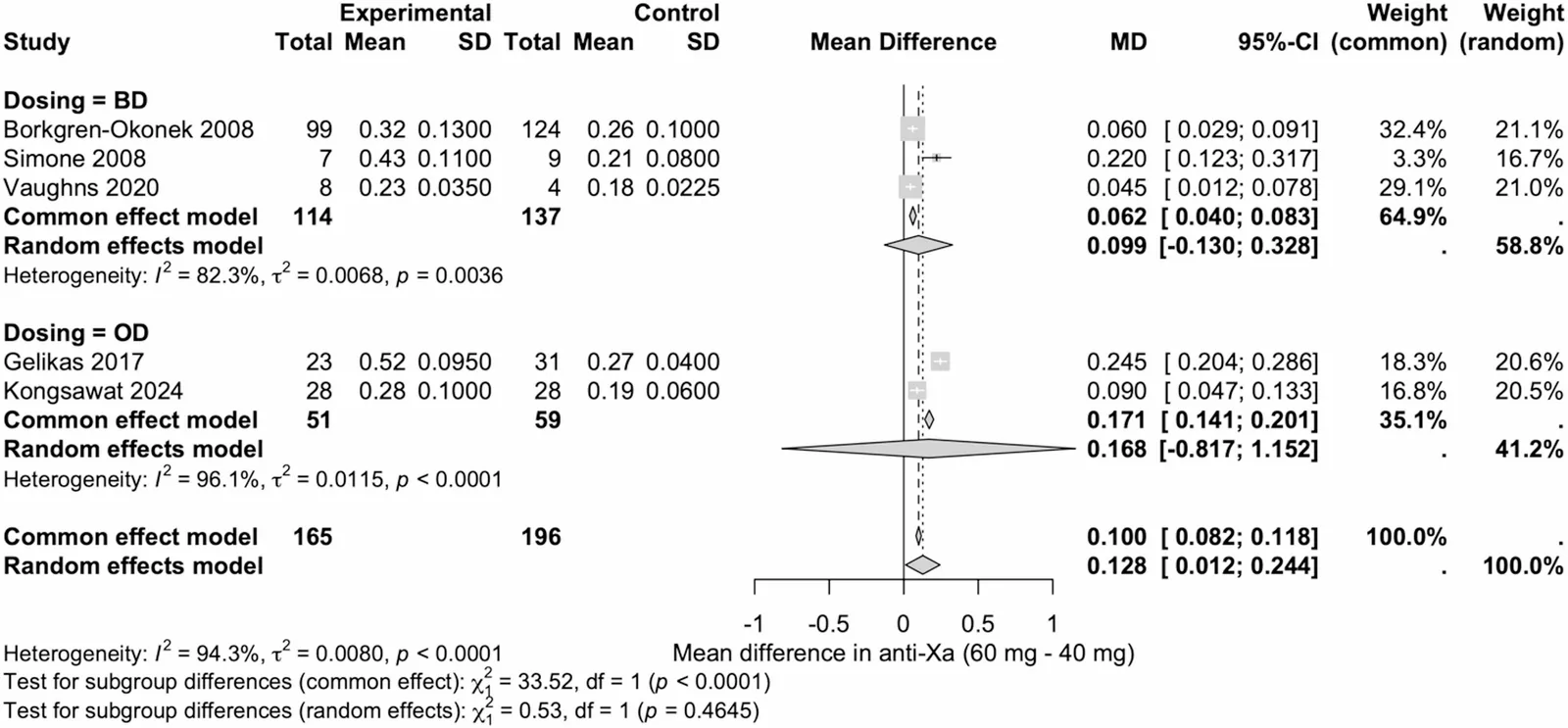
Analysis of Anti-Xa Levels Between Enoxaparin BD dosing and Enoxaparin OD dosing

There was no statistically significant difference between the once-daily and twice-daily dosing subgroups (χ² = 0.23, df = 2, *p* = 0.89; I² = 0%), indicating that dosing frequency did not significantly influence the pooled mean difference in anti-Xa levels (Fig. 7).

### Risk of Bleeding with Enoxaparin 40 mg or Enoxaparin 60 mg

Five studies reported bleeding outcomes across 575 patients (305 receiving lower-dose and 270 receiving higher-dose enoxaparin). A total of nine bleeding events were recorded: six in the lower-dose arm and three in the higher-dose arm. No fatal bleeding events were reported across any included study. The distribution of events is presented in Table 3 below. Formal meta-analysis was not performed as the combined sample was underpowered to produce clinically interpretable estimates.

#### Risk of a Venous Thromboembolic Event (VTE) with Enoxaparin 40 mg or Enoxaparin 60 mg

Six studies reported VTE outcomes across 631 patients (333 receiving lower-dose and 298 receiving higher-dose enoxaparin). A single VTE event was recorded across all included studies, occurring in the lower-dose arm of Borkgren-Okonek 2008. Follow-up duration for VTE surveillance was defined in only three of six studies, ranging from 10 days to three months. In three studies follow-up was limited to the perioperative inpatient period with no defined post-discharge VTE surveillance [28–30]. The distribution of events and follow-up duration for each study are presented in Table 3 below. Formal meta-analysis was not performed given the single event identified.

### Quality Assessment

Risk of bias was independently assessed by two coauthors (C.K. and M.A.). Both RCTs were rated Some Concerns on RoB 2 assessment. Steib 2015 had concerns in Domains 1, 2, and 3 relating to allocation concealment, open-label design, and exclusion of 18% of patients from per-protocol analysis; Domains 4 and 5 were Low Risk. Kongsawat 2024 had concerns solely in Domain 5 due to absence of trial registration and a pre-specified analysis plan, with all other domains rated Low Risk. The RoB 2 traffic light summary is presented in Supplementary Fig. 2. Observational studies were assessed using the Newcastle-Ottawa Scale (NOS); scores ranged from six to eight with a median of six. Full quality assessments are presented in Table 4 and a GRADE summary of findings for the primary outcome in Table 5.

## Discussion

This systematic review and meta-analysis integrated data from ten studies comprising 1,069 enrolled patients, of whom 1,016 contributed to the primary meta-analysis. The principal finding was that there was no significant difference in the proportion of patients achieving prophylactic anti-factor Xa levels between lower (30/40 mg) and higher (60 mg) enoxaparin regimens. Across the studies included in this meta-analysis, enoxaparin dosing strategies varied considerably, underscoring the absence of a uniform approach to thromboprophylaxis after MBS. Several investigations employed BMI- or weight-adjusted regimens, aiming to individualize anticoagulant dosing in recognition of the altered pharmacokinetics and increased thrombotic risk associated with severe obesity. In contrast, other studies used fixed-dose protocols, comparing standard with higher doses or once-daily with twice-daily administration, often based on local practice patterns or pharmacokinetic rationale. This variation highlights persistent uncertainty regarding the optimal prophylactic regimen in this setting. Overall, these findings reflect ongoing efforts to balance efficacy, safety, and practicality in the design of thromboprophylaxis protocols for patients undergoing MBS. Our findings mirror previous surveys of bariatric surgeons, which reported wide variation in dosing practices and duration of pharmacological prophylaxis [31]. This inconsistency reflects the absence of definitive evidence and the lack of consensus across major guideline bodies [8, 10, 13, 32]. Tables 1 and 3 summarises the different dosing regimens and proportion of doses that achieved prophylaxis.

The primary meta-analysis demonstrated no statistically significant difference in the proportion of patients achieving prophylactic anti-Xa targets between higher and lower enoxaparin dosing regimens. However, the confidence interval of 0.91–1.22 is wide and fully consistent with clinically meaningful benefit or harm in either direction; this result does not support a conclusion of equivalence. Substantial heterogeneity was observed (I² = 58.2%), reflecting genuine clinical and methodological variability across included studies in terms of dosing frequency, anti-Xa target ranges, patient population, and study design. The sensitivity analysis excluding the adolescent cohort of Vaughns 2020 produced a consistent result (RR 1.07, 95% CI 0.92–1.24; I² = 57.1%), confirming that residual heterogeneity is attributable to variability across the adult studies rather than the inclusion of the adolescent population. Taken together, the available pharmacodynamic evidence is inconclusive and insufficient to identify a superior dosing strategy. These findings underscore the need for large, adequately powered, multicentre randomised controlled trials with prospectively defined clinical endpoints to establish optimal enoxaparin dosing for VTE prophylaxis in patients undergoing MBS.

Although pooled analysis did not demonstrate a statistically significant difference in the proportion of patients achieving target anti-Xa levels, subgroup analyses suggested that higher doses (60 mg) were associated with significantly higher mean anti-Xa concentrations [33–36]. This effect was most pronounced in the once-daily dosing studies [29, 37], where a clear dose–response relationship was evident. In contrast, the twice-daily regimens demonstrated no significant difference in mean anti-Xa levels between 40 mg and 60 mg groups [28, 30, 38], possibly reflecting more stable pharmacokinetics with split dosing. Importantly, the largest included study (Borkgren-Okonek et al.) found no difference in mean anti-Xa levels between the two groups [38], which may have diluted the overall pooled effect.

Bleeding risk is a key consideration when escalating anticoagulant dosage. Descriptive analysis of bleeding outcomes across five studies showed nine events in total: six in the lower-dose arm and three in the higher-dose arm, with no fatal events reported [29, 39]. This finding aligns with previous systematic reviews, which concluded that while higher or weight-adjusted dosing may achieve more consistent anti-Xa levels, it does not necessarily increase bleeding risk [40, 41]. However, given that the included studies were underpowered to detect differences in clinical bleeding outcomes, these results should be interpreted with caution. VTE and bleeding outcomes were not formally pooled in this review due to critically low event rates, a single VTE event was recorded across 631 patients and the available data do not permit conclusions regarding comparative clinical safety between dosing regimens [28–30, 37]. The variability in follow-up duration across studies, ranging from the immediate postoperative period to three months may have contributed to under detection of VTE events, particularly in trials focused primarily on pharmacodynamic outcomes rather than clinical surveillance. This highlights the uncertainty of whether achieving higher anti-Xa levels correlates with meaningful clinical benefit. VTE rates after MBS remain relatively low (0.3–3%), but the clinical significance of even rare events must not be underestimated, as pulmonary embolism remains a leading cause of perioperative mortality in this cohort [12, 42, 43].

A 2024 meta-analysis of 2.73 million patients reported pooled VTE incidences of 0.15% in-hospital and ~ 0.5% at 30 days, with most events (≈ 60%) occurring post-discharge; rates were lower in recent studies and non–North American cohorts, and higher following open procedures [44]. Registry data from MBSAQIP similarly show 30-day VTE rates of ~ 0.3–0.4%, including revisional gastric bypass cases [45, 46]. While guidelines classify patients undergoing MBS as high risk and support pharmacological prophylaxis, they also emphasise individualised duration and dosing [47]. Risk models suggest extended prophylaxis is most appropriate when predicted post-discharge VTE risk exceeds 0.4–1.0% (e.g., prior VTE, heart failure, dyspnoea, prolonged surgery) [42]. Overall, contemporary evidence suggests population-level risk may be overestimated, yet in the absence of precise guidance, practice variation and broad prescribing persist.

Anti-Xa monitoring can guide individual dose adjustment but is impractical for routine use due to strict timing requirements and limited availability outside specialist centres [15, 18]. Most studies (*n* = 7) collected samples 3–5 h after the third enoxaparin dose, while two measured levels after the second dose to allow inpatient review and dose adjustment before discharge [33, 34]. 

The optimal LMWH dose for VTE prophylaxis in MBS remains debated. Weight-based dosing, pharmacist oversight, and anti-Xa monitoring are possible strategies to ensure adequate thromboprophylaxis [34]. Although DOACs offer convenience and favourable safety profiles elsewhere, uncertainty regarding absorption and pharmacokinetics after MBS means they are not currently recommended [48]. These findings underscore the ongoing dilemma of whether thromboprophylaxis should be prescribed using fixed regimens, BMI-stratified regimens, or fully weight-adjusted dosing [20].

### Limitations

This review has several limitations. Most included studies were small, single-institution observational cohorts; only two were randomised controlled trials, limiting causal inference and generalisability. Heterogeneity in study design, dosing protocols, and follow-up duration restricts the external validity of pooled estimates. Retrospective designs introduced selection and information bias, and follow-up for VTE was often short or poorly defined.

A fundamental limitation is that anti-Xa target attainment is a pharmacodynamic surrogate endpoint whose clinical validity in patients undergoing MBS has not been prospectively validated. The assumption that achieving 0.2–0.4 IU/mL reduces symptomatic VTE in this population is biologically plausible but unproven. Future trials should include clinical VTE events and bleeding as co-primary endpoints.

Our requirement for two enoxaparin dose arms excluded potentially informative single-arm pharmacokinetic studies. Pooling of the adolescent cohort (Vaughns 2020) with adult populations represents a source of unresolved clinical heterogeneity, and non-English publications may remain underrepresented despite no language restriction. Trial Sequential Analysis confirmed the required information size of 1,570 patients was not met, with the Z-curve remaining within the inconclusive zone throughout. Current evidence is therefore insufficient to draw definitive conclusions. Future research should prioritise large, multicentre randomised trials with standardised protocols and prospectively defined endpoints.

## Conclusion

These findings highlight that there is a lack of high-quality evidence and a wide variety of practice regarding thromboprophylaxis in patients with obesity undergoing MBS. The ideal prophylactic agent and dosage continue to be debated. Despite the advancement in treatment for obesity, no adequately powered RCT has been conducted to determine the optimal agent, dose, or duration of anticoagulant prophylaxis for VTE prevention in patients undergoing MBS, and the field remains reliant on expert consensus and institutional practice in the absence of robust evidence.

## Supplementary Information

Below is the link to the electronic supplementary material.

Supplementary Material 1 (DOCX 150 KB)

Supplementary Material 2 (PDF 49.8 KB)

## Data Availability

Yes. This study used data derived from previously published studies. All data analysed in this systematic review and meta-analysis are available within the included articles and their supplementary materials.

## Appendix

**Table 1:** Study Characteristics N/S = not specified, BMI = body mass index, RCT = randomised controlled trial

Author	Year	Location	Study Type	Total No. of patients	Dose of enoxaparin	Patients per arm	Patients achieving prophylaxis	Duration of treatment (days)
Borkgren-Okonek	2008	USA	Prospective	223	40 mg BD (BMI < 50 kg/m^2^)	124	86	10
					60 mg BD (BMI ≥ 50 kg/m^2^)	99	67	
Gelikas	2017	Israel	Prospective	54	40 mg OD	31	25	N/S
					60 mg OD	23	21	
Karas	2021	USA	Prospective	105	40 mg BD (BMI < 50 kg/m^2^)	70	60	14–28
					60 mg BD (BMI ≥ 50 kg/m^2^)	35	29	
Kongsawat	2024	Thailand	RCT	56	40 mg OD	28	15	N/S
					60 mg OD	28	22	
O’Connor	2024	USA	Retrospective	203	40 mg BD (BMI < 50 kg/m^2^)	90	57	N/S
					60 mg BD (BMI ≥ 50 kg/m^2^)	113	76	
Rowan	2007	USA	Prospective	52	30 mg BD	19	1	N/S
					40 mg BD	33	5	
Simone	2008	USA	Prospective	40	40 mg BD	24	4	N/S
					60 mg BD	16	4	
Steib	2015	France	RCT	88	40 mg OD	44	6	10
					60 mg OD	44	25	
Steir	2020	Germany	Prospective	236	40 mg BD (BMI < 50 kg/m^2^)	124	71	N/S
					60 mg BD (BMI ≥ 50 kg/m^2^)	112	68	
Vaughns	2020	USA	Prospective	12	40 mg BD (BMI < 50 kg/m^2^)	4	4	3
					60 mg BD (BMI ≥ 50 kg/m^2^)	8	6

**Table 2:** Patient characteristics SD = standard deviation, N/S = not specified

Author	Male	Female	BMI kg/m^2^ (lower dose) Mean (SD)	BMI kg/m^2^ (higher dose) Mean (SD)
Borkgren-Okonek	55	168	44.3 ± 10.6	44.7 ± 10.1
Gelikas	18	36	42.27 ± 0.84	44.15 ± 1.25
Karas	16	89	N/S	N/S
Kongsawat	22	34	45.37 ± 7.63	45.58 ± 8.85
O’Connor	67	136	N/S	N/S
Rowan	11	41	41.7 ± 10.7	40.8 ± 9.1
Simone	4	36	48.8 ± 6.6	47.3 ± 6.6
Steib	15	73	49 ± 1	48 ± 1
Steir	70	166	42.94 ± 6.84	63.68 ± 9.91
Vaughns	2	10	44.1 ± 2.85	54.05 ± 1.98

**Table 3:** Primary and secondary outcomes *median, †mean, SD = standard deviation, N/S = not specified

Author	Primary outcome	Timing of Blood Sampling	Dose of enoxaparin	Proportion achieving prophylaxis	Mean Anti-Xa Level IU/mL (SD)	VTE (*n*)	Bleeding (*n*)
Borkgren-Okonek	To assess anti-Xa activity in the patient population	4 h after the third dose	40 mg BD (BMI < 50 kg/m^2^)	69.35%	0.26 ± 0.10	1	4
			60 mg BD (BMI ≥ 50 kg/m^2^)	67.68%	0.32 ± 0.13	0	1
Gelikas	Anti-Xa levels in each of the two groups	3–4 h after the third dose	40 mg OD	80.6%	0.27 ± 0.04	N/S	N/S
			60 mg OD	91.3%	0.515 ± 0.095	N/S	N/S
Karas	Percentage of patients within the prophylactic range	3–5 h after the second dose	40 mg BD (BMI < 50 kg/m^2^)	85.7%	N/S	N/S	N/S
			60 mg BD (BMI ≥ 50 kg/m^2^)	82.9%	N/S	N/S	N/S
Kongsawat	To evaluate the achieving prophylactic anti-Xa levels with different doses of enoxaparin	4 h post first dose	40 mg OD	53.57%	0.19 ± 0.06	0	N/S
			60 mg OD	78.57%	0.28 ± 0.10	0	N/S
O’Connor	To evaluate the adequacy of BMI-based VTE prophylaxis using anti-Xa levels	4 h after the second or third dose	40 mg BD (BMI < 50 kg/m^2^)	63.33%	N/S	N/S	N/S
			60 mg BD (BMI ≥ 50 kg/m^2^)	67.26%	N/S	N/S	N/S
Rowan	To characterise anti-Xa levels in bariatric surgery patients receiving prophylactic enoxaparin	4 h after the third dose	30 mg BD	9.1%	0.08	N/S	N/S
			40 mg BD	42.42%	0.15	N/S	N/S
Simone	To assess if dosing achieved target prophylaxis as assessed by Anti-Xa levels	4 h after the third dose	40 mg BD	44.44%	0.21 ± 0.08	0	1
			60 mg BD	57.14%	0.43 ± 0.11	0	0
Steib	To determine the optimal regimen of enoxaparin providing anti-factor Xa peak activity	3–5 h after the third dose	40 mg OD	13.64%	N/S	0	1
			60 mg OD	56.82%	N/S	0	2
Steir	To evaluate the adequacy of BMI-based VTE prophylaxis using anti-Xa levels	3–4 h after the third dose	40 mg BD (BMI < 50 kg/m^2^)	57.26%	N/S	0	0
			60 mg BD (BMI ≥ 50 kg/m^2^)	60.71%	N/S	0	0
Vaughns	To assess if dosing achieved target prophylaxis as assessed by Anti-Xa levels	4 h after the first dose	40 mg BD (BMI < 50 kg/m^2^)	100%	0.185 ± 0.0225	0	0
			60 mg BD (BMI ≥ 50 kg/m^2^)	75%	0.23 ± 0.035	0	0

**Table 4:** Quality Assessment of Included Studies NOS = Newcastle Observation Score, RoB2 = Risk of Bias 2

Author	Year	Study Design	Quality Tool	Score/Classification
Borkgren-Okonek	2008	Prospective nonrandomised cohort	NOS	6/9
Gelikas	2017	Prospective cohort	NOS	7/9
Karas	2021	Retrospective cohort	NOS	6/9
Kongsawat	2024	RCT	RoB2	Some concerns
O’Connor	2024	Retrospective cohort	NOS	6/9
Rowan	2007	Prospective nonrandomised cohort	NOS	6/9
Simone	2007	Prospective nonrandomised cohort	NOS	6/9
Steib	2015	RCT	RoB2	Some concerns
Steir	2015	Prospective systematic cohort	NOS	8/9
Vaughns	2020	Prospective cohort (adolescents)	NOS	6/9

**Table 5:** GRADE Summary of Findings

Outcome	Studies (*n*)	Patients (*n*)	Risk of bias	Inconsistency	Indirectness	Imprecision	GRADE certainty
Anti-Xa target attainment	10	1,016	Serious¹	Moderate (I²=53.6%)²	Not serious	Serious³	LOW ⊕⊕○○

## References

[1] Abdollahi M, Cushman M, Rosendaal FR. Obesity: risk of venous thrombosis and the interaction with coagulation factor levels and oral contraceptive use. Thromb Haemost. 2003;89(03):493–8. 12624633

[2] Allman-Farinelli MA, editor. editor Obesity and venous thrombosis: a review. Seminars in thrombosis and hemostasis. © Thieme Medical; 2011. 10.1055/s-0031-1297369 22198855

[3] Stein PD, Matta F. Pulmonary embolism and deep venous thrombosis following bariatric surgery. Obes Surg. 2013;23:663–8. 23404239 10.1007/s11695-012-0854-2

[4] Cushman M, editor. editor Epidemiology and risk factors for venous thrombosis. Seminars in hematology. Elsevier; 2007. 10.1053/j.seminhematol.2007.02.004 PMC2020806 17433897

[5] Aminian A, Andalib A, Khorgami Z, Cetin D, Burguera B, Bartholomew J, et al. Who should get extended thromboprophylaxis after bariatric surgery? a risk assessment tool to guide indications for post-discharge pharmacoprophylaxis. Ann Surg. 2017;265(1):143–50. 28009739 10.1097/SLA.0000000000001686

[6] Winegar DA, Sherif B, Pate V, DeMaria EJ. Venous thromboembolism after bariatric surgery performed by Bariatric Surgery Center of Excellence Participants: analysis of the Bariatric Outcomes Longitudinal Database. Surg Obes Relat Dis. 2011;7(2):181–8. 21421182 10.1016/j.soard.2010.12.008

[7] Altieri MS, Yang J, Hajagos J, Spaniolas K, Park J, Gasparis AP, et al. Evaluation of VTE prophylaxis and the impact of alternate regimens on post-operative bleeding and thrombotic complications following bariatric procedures. Surg Endosc. 2018;32:4805–12. 29766305 10.1007/s00464-018-6231-z

[8] Aminian A, Vosburg RW, Altieri MS, Hinojosa MW, Khorgami Z. The American Society for Metabolic and Bariatric Surgery (ASMBS) updated position statement on perioperative venous thromboembolism prophylaxis in bariatric surgery. Surg Obes Relat Dis. 2022;18(2):165–74. 34896011 10.1016/j.soard.2021.10.023

[9] Hamadi R, Marlow CF, Nassereddine S, Taher A, Finianos A. Bariatric venous thromboembolism prophylaxis: an update on the literature. Expert Rev Hematol. 2019;12(9):763–71. 31219356 10.1080/17474086.2019.1634542

[10] Arcelus JI, Gouin-Thibault I, Samama CM. European guidelines on peri-operative venous thromboembolism prophylaxis: first update.: Chap. 10: Surgery in the obese patient. Eur J Anaesthesiol | EJA. 2024;41(8):607–11. 38957028 10.1097/EJA.0000000000002000

[11] Birkmeyer NJ, Finks JF, Carlin AM, Chengelis DL, Krause KR, Hawasli AA, et al. Comparative effectiveness of unfractionated and low-molecular-weight heparin for prevention of venous thromboembolism following bariatric surgery. Arch Surg. 2012;147(11):994–8. 23165612 10.1001/archsurg.2012.2298

[12] Guzman-Pruneda FA, Garcia A, Crum RW, Chen T, Krikhely A, Bessler M. Extended Post Discharge Prophylaxis for Venous Thromboembolism Prevention After Bariatric Surgery. Obes Surg. 2024;34(4):1217–23. 38409623 10.1007/s11695-024-07100-z

[13] NICE. Venous thromboembolism in over 16s: reducing the risk of hospital-acquired deep vein thrombosis or pulmonary embolism. editor. United Kingdom: National Institute for Health and Care Excellence; 2019. NICE. 32924386

[14] Zee AA, van Lieshout K, van der Heide M, Janssen L, Janzing HM. Low molecular weight heparin for prevention of venous thromboembolism in patients with lower-limb immobilization. Cochrane Database Syst Rev. 2017. 10.1002/14651858.CD006681.pub4. 28780771 PMC6483324

[15] Paige JT, Gouda BP, Gaitor-Stampley V, Scalia PG, Klainer TE, Raum WJ, et al. No correlation between anti-factor Xa levels, low-molecular-weight heparin, and bleeding after gastric bypass. Surg Obes Relat Dis. 2007;3(4):469–75. 17567541 10.1016/j.soard.2006.12.012

[16] Nutescu EA, Spinier SA, Wittkowsky A, Dager WE. Anticoagulation: low-molecular-weight heparins in renal impairment and obesity: available evidence and clinical practice recommendations across medical and surgical settings. Ann Pharmacother. 2009;43(6):1064–83. 19458109 10.1345/aph.1L194

[17] Sacha GL, Greenlee KM, Ketz JM. The use of anti-factor Xa monitoring in a selection of patients receiving enoxaparin at a large academic medical center. J Thromb Thrombolysis. 2016;42(4):479–85. 27256341 10.1007/s11239-016-1384-x

[18] Egan G, Ensom MH. Measuring anti–factor Xa activity to monitor low-molecular-weight heparin in obesity: a critical review. Can J Hosp Pharm. 2015;68(1):33. 25762818 10.4212/cjhp.v68i1.1423 PMC4350497

[19] Phyo WW, Deodhar K, Chang A, Blair M, Boyd AN, Geik C. Prophylactic enoxaparin dosing and anti-Xa levels in medicine patients with obesity. J Pharm Technol. 2025. 10.1177/87551225251328255. 40170754 PMC11955971

[20] Ikesaka R, Delluc A, Le Gal G, Carrier M. Efficacy and safety of weight-adjusted heparin prophylaxis for the prevention of acute venous thromboembolism among obese patients undergoing bariatric surgery: a systematic review and meta-analysis. Thromb Res. 2014;133(4):682–7. 24508449 10.1016/j.thromres.2014.01.021

[21] Liberati A, Altman DG, Tetzlaff J, Mulrow C, Gøtzsche PC, Ioannidis JP, et al. The PRISMA statement for reporting systematic reviews and meta-analyses of studies that evaluate health care interventions: explanation and elaboration. J Clin Epidemiol. 2009;62(10):e1–34. 19631507 10.1016/j.jclinepi.2009.06.006

[22] Trial Sequential Analysis. Version 0.9.5.10 Beta ed. Copenhagen: Copenhagen Trial Unit, Centre for Clinical Intervention Research. Copenhagen University Hospital – Rigshospitalet; 2021.

[23] Sterne JA, Savović J, Page MJ, Elbers RG, Blencowe NS, Boutron I, et al. RoB 2: a revised tool for assessing risk of bias in randomised trials. BMJ. 2019. 10.1136/bmj.l4898. 31462531

[24] Sterne JAC, Savović J, Page MJ, Elbers RG, Blencowe NS, Boutron I, et al. RoB 2: a revised tool for assessing risk of bias in randomised trials. BMJ. 2019;366:l4898. 31462531 10.1136/bmj.l4898

[25] Peterson J, Welch V, Losos M, Tugwell P. The Newcastle-Ottawa scale (NOS) for assessing the quality of nonrandomised studies in meta-analyses. Ottawa: Ottawa Hospital Research Institute; 2011.

[26] Stang A. Critical evaluation of the Newcastle-Ottawa scale for the assessment of the quality of nonrandomized studies in meta-analyses. Eur J Epidemiol. 2010;25(9):603–5. 20652370 10.1007/s10654-010-9491-z

[27] Guyatt GH, Oxman AD, Vist GE, Kunz R, Falck-Ytter Y, Alonso-Coello P, et al. GRADE: an emerging consensus on rating quality of evidence and strength of recommendations. BMJ. 2008;336(7650):924–6. 18436948 10.1136/bmj.39489.470347.AD PMC2335261

[28] Simone EP, Madan AK, Tichansky DS, Kuhl DA, Lee MD. Comparison of two low-molecular-weight heparin dosing regimens for patients undergoing laparoscopic bariatric surgery. Surg Endosc. 2008;22(11):2392–5. 18594915 10.1007/s00464-008-9997-6

[29] Kongsawat K, Chaivanijchaya K, Pakul F, Joradol S, Kachornvitaya P, Boonchaya-Anant P, et al. Comparison of enoxaparin 40 mg versus 60 mg dosage for venous thromboprophylaxis in patients undergoing bariatric surgery: A randomized controlled trial. Asian J Surg. 2024;47(7):2985–90. 38514281 10.1016/j.asjsur.2024.02.095

[30] Vaughns JD, Ziesenitz VC, Williams EF, Nadler EP, Mikus G, van den Anker J. Prophylactic use of enoxaparin in adolescents during bariatric surgery—a prospective clinical study. Obes Surg. 2020;30(1):63–8. 31463801 10.1007/s11695-019-04135-5 PMC7375856

[31] Giannopoulos S, Motamedi SMK, Athanasiadis DI, Clapp B, Lyo V, Ghanem O, et al. Venous thromboembolism (VTE) prophylaxis after bariatric surgery: a national survey of MBSAQIP director practices. Surg Obes Relat Dis. 2023;19(8):799–807. 36717309 10.1016/j.soard.2022.12.038 PMC11651302

[32] DeMaria EJ, Kothari SN, Rogers AM. VTE prevention in bariatric surgery: from where have we come, and how did we get here? Surg Obes Relat Dis. 2022;18(2):175–6. 34920965 10.1016/j.soard.2021.10.022

[33] Steele KE, Canner J, Prokopowicz G, Verde F, Beselman A, Wyse R, et al. The EFFORT trial: Preoperative enoxaparin versus postoperative fondaparinux for thromboprophylaxis in bariatric surgical patients: a randomized double-blind pilot trial. Surg Obes Relat Dis. 2015;11(3):672–83. 25620436 10.1016/j.soard.2014.10.003

[34] Karas LA, Nor Hanipah Z, Cetin D, Schauer PR, Brethauer SA, Daigle CR, et al. Assessment of empiric body mass index-based thromboprophylactic dosing of enoxaparin after bariatric surgery: evidence for dosage adjustment using anti-factor Xa in high-risk patients. Surg Obes Relat Dis. 2021;17(1):153–60. 33046419 10.1016/j.soard.2020.08.016

[35] Raftopoulos I, Martindale C, Cronin A, Steinberg J. The effect of extended post-discharge chemical thromboprophylaxis on venous thromboembolism rates after bariatric surgery: a prospective comparison trial. Surg Endosc. 2008;22(11):2384–91. 18622558 10.1007/s00464-008-0031-9

[36] Cossu ML, Pilo L, Piseddu G, Tilocca PL, Cossu F, Noya G. Prophylaxis of venous thromboembolism in bariatric surgery. Chirurgia Italiana. 2007;59(3):331–5. 17663372

[37] Gelikas S, Eldar SM, Lahat G. Anti-factor Xa levels in patients undergoing laparoscopic sleeve gastrectomy: 2 different dosing regimens of enoxaparin. Surg Obes Relat Dis. 2017;13(10):1753–9. 28918992 10.1016/j.soard.2017.07.027

[38] Borkgren-Okonek MJ, Hart RW, Pantano JE, Rantis PC Jr, Guske PJ, Kane JM Jr, et al. Enoxaparin thromboprophylaxis in gastric bypass patients: extended duration, dose stratification, and antifactor Xa activity. Surg Obes Relat Dis. 2008;4(5):625–31. 18261965 10.1016/j.soard.2007.11.010

[39] Steib A, Degirmenci S-E, Junke E, Asehnoune K, Figier M, Pericard C, et al. Once versus twice daily injection of enoxaparin for thromboprophylaxis in bariatric surgery: effects on antifactor Xa activity and procoagulant microparticles. A randomized controlled study. Surg Obes Relat Dis. 2016;12(3):613–21. 26686309 10.1016/j.soard.2015.08.505

[40] Brotman DJ, Shihab HM, Prakasa KR, Kebede S, Haut ER, Sharma R, et al. Pharmacologic and mechanical strategies for preventing venous thromboembolism after bariatric surgery: a systematic review and meta-analysis. JAMA Surg. 2013;148(7):675–86. 23754086 10.1001/jamasurg.2013.72

[41] Becattini C, Agnelli G, Manina G, Noya G, Rondelli F. Venous thromboembolism after laparoscopic bariatric surgery for morbid obesity: clinical burden and prevention. Surg Obes Relat Dis. 2012;8(1):108–15. 22014482 10.1016/j.soard.2011.09.005

[42] Aminian A, Andalib A, Khorgami Z, Cetin D, Burguera B, Bartholomew J, et al. Who should get extended thromboprophylaxis after bariatric surgery? Ann Surg. 2017;265(1):143–50. 28009739 10.1097/SLA.0000000000001686

[43] Zhao Y, Ye Z, Lin J, Zhang Z, Tian P, Zhang Z, et al. Efficacy and Safety of Pharmacoprophylaxis for Venous Thromboembolism in Patients Undergoing Bariatric Surgery: a Systematic Review and Meta-analysis. Obes Surg. 2022;32(5):1701–18. 35296968 10.1007/s11695-021-05825-9

[44] El Ansari W, El-Menyar A, El-Ansari K, Al-Ansari A, Lock M. Cumulative Incidence of Venous Thromboembolic Events In-Hospital, and at 1, 3, 6, and 12 Months After Metabolic and Bariatric Surgery: Systematic Review of 87 Studies and Meta-analysis of 2,731,797 Patients. Obes Surg. 2024;34(6):2154–76. 38602603 10.1007/s11695-024-07184-7 PMC11127857

[45] Leslie ZD, Quinn CM, Ikramuddin S, Wise ES. Venous Thromboembolism After Bariatric Surgery: An Analysis of Predictors and Trends in Two Large Datasets. The American Surgeon^TM^. 2025;91(11):1898–1905. 10.1177/00031348251339525. 40340475

[46] Economopoulos KP, Szoka N, Eckhouse SR, Chumakova-Orin M, Kuchibhatla M, Merchant J, et al. Venous thromboembolic events following revisional gastric bypass: an analysis of the MBSAQIP database from 2015 to 2019 using propensity matching. Obes Surg. 2015;34(12):4358–68. 10.1007/s11695-024-07511-y 39349920

[47] Arcelus JI, Gouin-Thibault I, Samama CM. European guidelines on peri-operative venous thromboembolism prophylaxis: first update.: Chap. 10: Surgery in the obese patient. Eur J Anaesthesiol | EJA. 2024;41(8). 10.1097/EJA.0000000000002000 38957028

[48] Li A, Eshaghpour A, Tseng EK, Douketis JD, Anvari M, Tiboni M, et al. Weight-adjusted tinzaparin for venous thromboembolism prophylaxis in bariatric surgery patients weighing 160 kg or more. Thromb Res. 2021;198:1–6. 33246191 10.1016/j.thromres.2020.11.021

