# Supplementary material for: Prophylactic Enoxaparin Dosing After Bariatric Surgery: A Systematic Review and Meta-Analysis of Pharmacodynamic Efficacy and Safety
Source: Obes Surg. 2026 Jul 21;36(9):4899–913. doi: 10.1007/s11695-026-08825-9 (PMC13562119; doi:10.1007/s11695-026-08825-9)
Supplement: Supplementary file 1 [file 11695_2026_8825_MOESM2_ESM.pdf]

Unique ID	Experimental	Comparator	Outcome	D1	D2	D3	D4	D5	Overall
Steib 2015	60 mg	40 mg	Proportion Achieving Prophylaxis	!	!	+	+	+	!
Kongsawat 2024	60 mg	40 mg	Proportion Achieving Prophylaxis	+	+	+	+	!	!

+ Low risk

! Some concerns

- High risk

D1 Randomisation process

D2 Deviations from the intended interventions

D3 Missing outcome data

D4 Measurement of the outcome

D5 Selection of the reported result
