# Supplementary material for: Prophylactic Enoxaparin Dosing After Bariatric Surgery: A Systematic Review and Meta-Analysis of Pharmacodynamic Efficacy and Safety
Source: Obes Surg. 2026 Jul 21;36(9):4899–913. doi: 10.1007/s11695-026-08825-9 (PMC13562119; doi:10.1007/s11695-026-08825-9)
Supplement: Supplementary file 2 [file 11695_2026_8825_MOESM1_ESM.docx]

**Supplementary Appendix 1**

**Full Database-Specific Search Strings**

The following search strategies were applied to each database. All searches were conducted up to 31st July 2025. Search strings were adapted to the syntax and controlled vocabulary of each database. The PubMed string served as the reference strategy from which all others were derived.

**1. PubMed (via NCBI, MeSH terms)**

Search interface: PubMed (https://pubmed.ncbi.nlm.nih.gov). Controlled vocabulary: Medical Subject Headings (MeSH). The [Mesh] tag was used to map terms to the MeSH thesaurus with explosion to narrower terms. Free-text synonyms were included in all concept blocks.

("Bariatric Surgery"[Mesh] OR "bariatric surgery" OR "obesity surgery" OR

"weight loss surgery" OR "Roux-en-Y" OR "gastric bypass" OR

"sleeve gastrectomy")

AND

("Low-Molecular-Weight Heparin"[Mesh] OR "LMWH" OR "enoxaparin")

AND

("Factor Xa"[Mesh] OR "anti-Xa" OR "anti-factor Xa" OR

"anti-Xa monitoring" OR "anti-Xa level*")

AND

("Venous Thromboembolism"[Mesh] OR "VTE" OR "deep vein thrombosis" OR

"DVT" OR "pulmonary embolism" OR "PE" OR "thromboprophylaxis")

**2. EMBASE (via Ovid, Emtree terms)**

Search interface: EMBASE (<https://www.embase.com>). The search was conducted via the direct Elsevier EMBASE interface. Term explosion to narrower concepts is applied automatically by this interface and the /exp tag is not required. Free-text synonyms were combined with Boolean operators within each concept block, and concept blocks were combined using AND.

('bariatric surgery' OR 'obesity surgery' OR 'weight loss surgery'

OR 'Roux-en-Y gastric bypass' OR 'gastric bypass' OR 'sleeve gastrectomy')

AND

('low molecular weight heparin' OR 'LMWH' OR 'enoxaparin')

AND

('anti-Xa' OR 'anti-factor Xa' OR 'anti-Xa monitoring' OR 'anti-Xa level')

AND

('venous thromboembolism' OR 'VTE' OR 'deep vein thrombosis' OR 'DVT'

OR 'pulmonary embolism' OR 'PE' OR 'thromboprophylaxis')

**3. Cochrane Library (via Cochrane Search Manager, MeSH terms)**

Search interface: Cochrane Library Search Manager (https://www.cochranelibrary.com). Controlled vocabulary: MeSH headings applied using the [mh "..."] tag. The Cochrane Central Register of Controlled Trials (CENTRAL), Cochrane Database of Systematic Reviews (CDSR), and the Database of Abstracts of Reviews of Effects (DARE) were searched simultaneously.

([mh "Bariatric Surgery"] OR "bariatric surgery" OR "obesity surgery" OR

"weight loss surgery" OR "Roux-en-Y" OR "gastric bypass" OR

"sleeve gastrectomy")

AND

([mh "Heparin, Low-Molecular-Weight"] OR "low molecular weight heparin"

OR "LMWH" OR "enoxaparin")

AND

([mh "Factor Xa"] OR "anti-Xa" OR "anti-factor Xa" OR

"anti-Xa monitoring" OR "anti-Xa level*")

AND

([mh "Venous Thromboembolism"] OR "VTE" OR "deep vein thrombosis" OR

"DVT" OR [mh "Pulmonary Embolism"] OR "pulmonary embolism" OR "PE" OR

"thromboprophylaxis")

**4. ClinicalTrials.gov (keyword search)**

Search interface: ClinicalTrials.gov (https://clinicaltrials.gov). ClinicalTrials.gov does not support MeSH explosion or Boolean field-level syntax in its standard search interface. The search was therefore conducted using keyword terms entered into the Conditions, Interventions, and Other Terms fields as described below. Results were filtered to include interventional and observational studies, with no restriction on recruitment status, in order to capture completed but unpublished trials.

**Conditions field:**

bariatric surgery OR obesity surgery OR sleeve gastrectomy OR gastric bypass

**Interventions field:**

enoxaparin OR low molecular weight heparin OR LMWH

**Other terms field:**

anti-Xa OR anti-factor Xa OR thromboprophylaxis OR venous thromboembolism

**5. Grey Literature**

In addition to the above database searches, the following sources were searched for grey literature and unpublished data:

**OpenGrey** (www.opengrey.eu): keyword search using 'bariatric surgery' AND 'enoxaparin' AND 'anti-Xa'.

**WHO International Clinical Trials Registry Platform (ICTRP)** (www.who.int/ictrp): keyword search using 'bariatric surgery' AND 'enoxaparin'.

**Conference proceedings**: abstracts from the American Society for Metabolic and Bariatric Surgery (ASMBS) annual scientific meeting were manually searched.

**Author contact**: authors of relevant trials identified through registry searches that had been completed but not yet published were contacted by email to request access to unpublished data. No additional datasets were obtained as the authors did not respond to us.

**Supplementary Figure 1:**

**Supplementary Figure 1.** Trial Sequential Analysis for the primary outcome of prophylactic anti-Xa target attainment comparing higher versus lower enoxaparin dosing regimens. The blue line represents the cumulative Z-curve. Red horizontal lines represent the O'Brien-Fleming adjusted monitoring boundaries (two-sided alpha = 0.05, power = 80%). The vertical line at n = 558 represents the point at which the required information size calculation was initiated. The required information size of 1,570 patients was not reached, and the Z-curve did not cross either boundary, indicating inconclusive evidence.

**Supplementary Figure 2:** Cochrane Risk of Bias 2 (RoB 2) assessment for the two randomised controlled trials included in the meta-analysis. D1: randomisation process; D2: deviations from intended interventions; D3: missing outcome data; D4: measurement of the outcome; D5: selection of the reported result. + Low risk; ! Some concerns; − High risk
